# Recreational music exposure and hearing health in young adults

**DOI:** 10.1007/s00405-024-08666-1

**Published:** 2024-05-13

**Authors:** Onur Ergun, Eda Cakmak, Asuman Alniacik

**Affiliations:** 1 Department of Otorhinolaryngology-Head and Neck Surgery, Baskent University Faculty of Medicine, Ankara, Turkey; 2 Department of Audiology, Baskent University Faculty of Medicine, Ankara, Turkey

**Keywords:** Recreational music, Sensorineural hearing loss, Noise-induced hearing loss, Extended-spectrum audiogram

## Abstract

**Objective:**

This study aimed to compare daily and total recreational music exposure levels and extended-spectrum audiogram results in young adults without pre-existing hearing problems.

**Design:**

The study included healthy volunteers aged 18–25 with no known ear disease or hearing loss. Participants completed a questionnaire, underwent otoscopic and tympanometric examinations, and determined preferred music volumes in an audiometry booth using calibrated music samples of their preferred genres. Hearing thresholds up to 16 kiloHertz (kHz) were measured. Daily music exposure for each participant was normalized to 8 h to calculate a time-weighted average of 8 h (TWA8). Total exposure (TE) was calculated by multiplying TWA8 by the number of years of music listening.

**Results:**

A total of 32.4% of participants had TWA8s above 65 dB. Their hearing thresholds at 125, 250, 500, and 16,000 Hz and the average of 125 Hz–8 kHz were significantly higher. Participants with TWA8s above 65 dB were also more prone to speaking loudly and experiencing communication difficulties on the phone. Those with a TE of more than 400 experienced significantly more speech discrimination difficulty in noisy environments and temporary hearing loss/tinnitus after exposure to loud music. Participants with a TE above 700 had worse thresholds at 4, 14, and 16 kHz frequencies, as well as 125–8000 Hz and 500–4000 Hz averages compared to those with a TE below 700.

**Conclusions:**

This study provides evidence that recreational music with much lower exposure levels than the universally accepted TWA8 of 85 dB could negatively impact hearing in healthy young adults. Therefore, maintaining a maximum TWA8 of 65 dB is recommended.

## Introduction

Recreational music exposure has gained significant popularity among young individuals [1–3], raising concerns about its potential impact on hearing health. While the link between occupational noise exposure and hearing loss has been extensively studied [4], the impact of recreational music on hearing remains relatively understudied [1, 5], with limited awareness among the general population [6, 7]. Given that any form of sound exposure could lead to progressive sensorineural hearing loss [2] which may become only apparent after many years or even decades, it is crucial to identify preventable causes and improve public health, and quality of life [7].

Studies examining the effect of music exposure on hearing have employed various methods to understand the extent of music exposure. Earlier studies have predominantly relied on self-reported questionnaires [1, 2, 6]. However, recently some researchers have adopted more objective approaches, such as utilizing pre-calibrated personal listening devices and asking participants to select their preferred music volume [3, 8]. While most studies used routine pure-tone audiometry for hearing evaluation, a few have utilized extended-spectrum audiograms to detect earlier signs of hearing loss [8].

This study aimed to investigate the relationship between recreational music exposure and sensorineural hearing loss in young adults without pre-existing hearing problems as objectively as possible using self-determined levels of their preferred music genres and extended-spectrum audiograms.

## Materials and methods

This study was approved by the Institutional Review Board and Ethics Committee (Project no: KA23/25). All participants were included after giving informed consent.

### Subjects

The subjects consisted of healthy volunteers aged between 18 and 25 years. To ensure the absence of active ear disease or hearing problems, a questionnaire was administered, covering topics, such as the use of hearing aids, known ear diseases, history of ear surgeries (except ventilation tube insertion), use of ototoxic drugs, and regular exposure to loud noise (excluding entertainment music more than once a week). Individuals who responded positively to any of these questions were excluded from the study.

### Questionnaire

In addition to demographic information and exclusion criteria, participants were retrospectively asked about their average daily listening time to music, the number of years they have been listening to music, preferred genres, the loudness of the environment during music listening, the type of listening device, and the presence of active noise canceling technology. Participants were also asked about their difficulty in understanding speech in quiet/noisy environments, and during phone conversations. Additionally, subjective symptoms, such as being warned for speaking loudly, experiencing ear pain after listening to loud music, sensitivity to sound, temporary hearing loss, or tinnitus were also asked.

### Calibration of the samples and detection of the preferred music level

An array of 1-min music samples representing different genres (Rock: Green Day-Boulevard of Broken Dreams; jazz: Frank Sinatra-Fly me to the moon; pop: Lady Gaga-Just dance; electro: Galantis-Runaway; classical: Ilyich Tchaikovsky-Swan Lake; hip-hop: Timbaland-The way I are) were prepared. These samples were played in the quiet audiometric test booth using a Bluetooth speaker (Soundcore Motion B, Anker, China). The volume levels were set at 6 increasing increments to cover a range of intensities. The booth was also equipped with a sound level meter (SLM; Type 2250, Hottinger Brüel & Kjær, Denmark) positioned at head level to record the average decibel (dB) value of all frequencies within the 20–20 kHz range (dBA). After obtaining the recordings, the average decibel for each volume level of the music genres was calculated for comparison.

On the test day, the participants were asked to determine their preferred music level (between volume levels 1 and 6) of their preferred genres in the test booth. Since the average decibels of each sample had been known at all volume levels, it was possible to identify the preferred decibel level for each participant based on their preferences.

### Hearing evaluation

Hearing thresholds between 125 Hz and 16 kiloHertz (kHz) and speech recognition scores (SRSs) were tested by an audiometer (Interacoustics AC40 clinical audiometry, Denmark) inside a sound-treated test room for all subjects.

### Calculating daily and total music exposures

The daily exposure to music for each participant was normalized to an equivalent of 8 h of continuous exposure, known as the Time-Weighted Average of 8 h (TWA8), using the following formula:

$${\text{TWA}}8 = 85 + 10\log_{10} \left( {T/8 \times 2^{{\left( {L - 85} \right)/3}} } \right).$$

In the formula, “*T*” represents the time in hours, and “*L*” represents the preferred music volume in decibels. Calculating the TWA8 scores, allowed for a fair comparison between participants.

The total music exposure of each participant (TE) was determined by multiplying their TWA8 with the number of years of music listening.

### Statistical analysis

Statistical analyses were conducted by IBM SPSS Statistics Version 25.0. Armonk. NY: IBM Corp. To evaluate the normality of distribution, Kolmogorov–Smirnov test was used. Mann–Whitney *U* test was used to examine the difference between pure-tone averages according to different cut-off values. Pearson chi-square test, continuity correction chi-square test, and Fisher’s exact test were used for categorical variables. The level of statistical significance was considered *p* < 0.05. *p < 0.05; **p < 0.01; ***p < 0.001.

## Results

The mean age of the 182 participants was 21.5 years, with 62.4% being female. The average hearing threshold for frequencies between 125 and 8000 Hz was 5.9 dB, ranging from − 1.4 to 28.2 dB. Speech discrimination scores were 88% and above. Among the participants, 50.5% reported no regular exposure to loud noises, such as concerts, discos, or shooting, while 17.6% reported one exposure, and 26.4% reported two exposures per month.

Regarding audio devices, 50% preferred earphones, 41% preferred headphones, and the remaining participants used speakers while listening to music. Approximately 33.5% of the participants listened to music in quiet environments. Additionally, 61.5% reported slightly noisy environments, while 4.9% reported moderately noisy environments while listening to music. Active noise cancelation technology was utilized by 44% of all participants.

Pop music was the most preferred genre followed by rock, classical, hip hop, jazz, and electro. During the calibration of 1-min samples of different music genres, it was found that classical music had the lowest dB values, while electro had the highest dB values at similar volume settings. After accounting for the participants’ volume preferences, there was up to 12 dB of difference between the preferred listening levels of different genres (Table 1).

**Table 1:** The mean measured decibel levels of various music genres at different volume levels (vol. 1–6)

	Vol. 1	Vol. 2	Vol. 3	Vol. 4	Vol. 5	Vol. 6	Mean (SD)
Classic	30.6	42.3	50.1	58	66.2	69.9	58 (8.9)
Jazz	38.4	50.3	57.9	66	73.7	77.8	64 (8.7)
Rock	44.1	56	63.9	72	80	83.6	68.3 (8.7)
Pop	45.9	57.9	65.5	73.7	81.5	84	69 (9)
Hip hop	46.3	57.9	66	74	81.6	84.4	69.6 (8.2)
Electro	49.2	61.1	68.8	76,8	82.2	86.2	70.3 (6.9)

The mean preferred listening decibel of each genre and its standard deviation (SD) is at the right column

After conducting TWA8 calculations, the participants were grouped based on cut-offs of 55 dB, 60 dB, 65 dB, and 70 dB. Due to the distribution of TWA8 scores (mean 61.7 ± SD 8.7 dB) and the limited number of subjects, the subgroups with the 55 dB and 70 dB thresholds became too small to draw reliable conclusions.

The participants with TWA8 scores higher than 60 dB, had significantly worse hearing thresholds at 125 Hz** than the participants with lower exposure. For those with TWA8s above 65 dB, thresholds at 125***, 250**, 500**, and 16 kHz** (Fig. 1) were significantly worse (Table 2). When the average hearing thresholds were examined, the most obvious difference was again in the TWA = 65 dB group. The differences were also significant for the 125–8 kHz* average and for the extended-spectrum averages (8–16 kHz*, 12.5–16 kHz**, and 14–16 kHz**). Participants with TWA8 values above 60 dB experienced significantly more difficulty in communicating on the phone. Additionally, “those with TWA8 ≥ 65 received significantly more warnings for speaking loudly” (Table 3).

**“Fig. 1:**
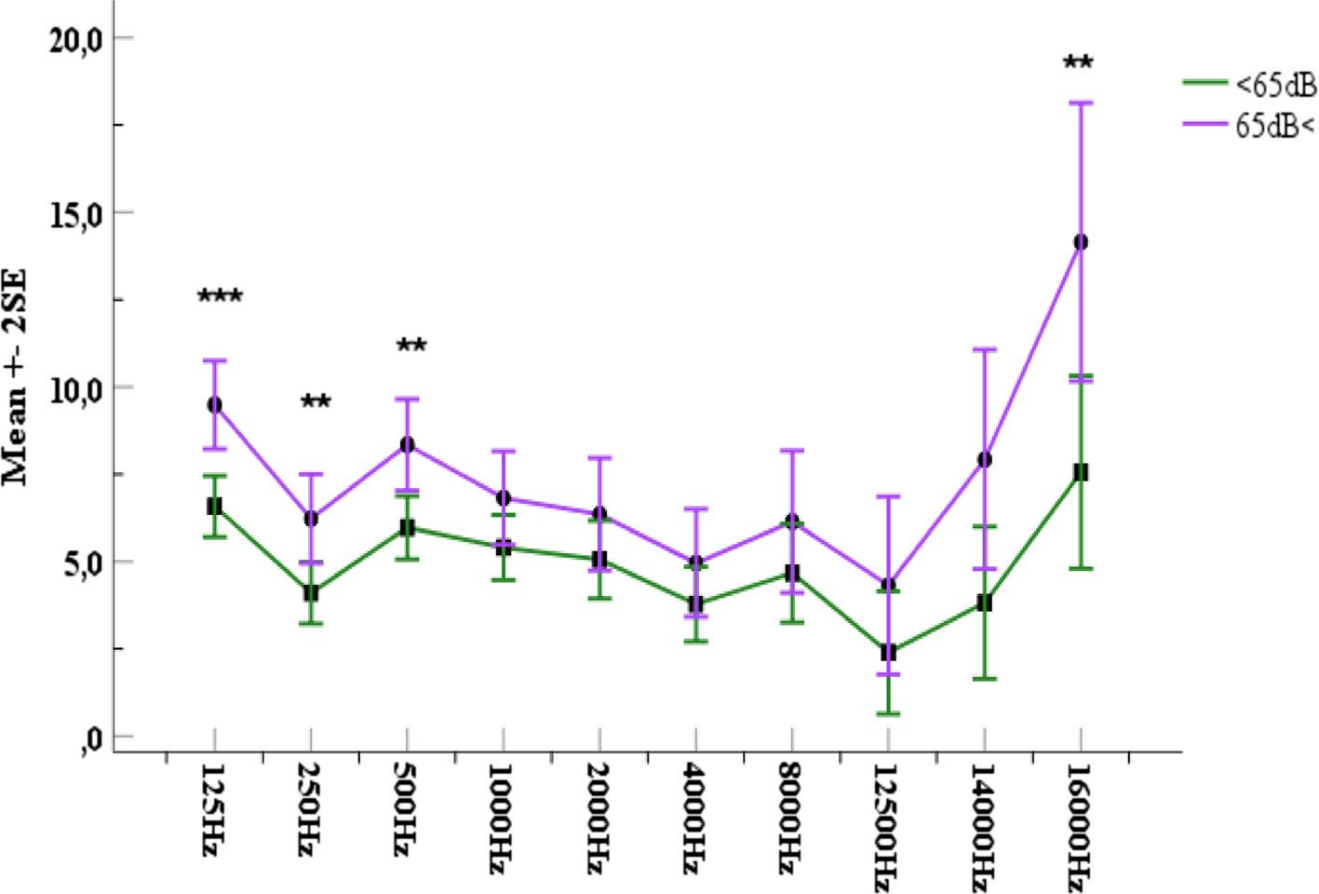
The extended-spectrum hearing thresholds are presented as mean ± 2 standard errors (SE). The green line represents the threshold values of the participants exposed to daily music for less than 8 h of continuous exposure to 65 decibels (dB). Please note that the participants with higher than 65 dB daily music exposure (the purple line) have significantly worse thresholds at both low and high frequencies. *p < 0.05, **p < 0.01, ***p < 0.001. *Hz* hertz

**Table 2:** The distribution of mean hearing thresholds among participants with varying daily music exposures (adjusted to 8 h of continuous exposure) is provided

	< 60 dB *n* = 78	≥ 60 dB *n* = 104	< 65 dB *n* = 123	≥ 65 dB n = 59
125 Hz	6.6*	8.2*	6.6*	9.5*
250 Hz	4.1	5.3	4.1*	6.2*
500 Hz	6.4	7	6*	8.3*
1 kHz	5.2	6.3	5.4	6.8
2 kHz	5.2	5.7	5.1	6.4
4 kHz	4.2	4.2	3.8	5
8 kHz	4.5	5.6	4.7	6.1
12.5 kHz	2.5	3.4	2.4	4.3
14 kHz	3.4	6.5	3.8	7.9
16 kHz	7.3	11.5	7.6*	14.2*

*Hz* hertz, *dB* decibel

The letter “*n*” represents the number of subjects, and “*” indicates statistical significance (*p* < 0.05)

**Table 3:** The subjective complaints of participants above various cut-off values (based on their daily music exposure levels adjusted to an 8-h continuous exposure) were compared to others who have less exposure

	≥ 60 dB	≥ 65 dB
Being warned for speaking loudly	0.34	0.01*
Speech discrimination difficulty in silent environments	0.66	0.86
Speech discrimination difficulty in noisy environments	0.7	0.86
Difficulty in communicating on the phone	0.04*	0.04*
Sound sensitivity after loud music	0.1	0.74
Temporary hearing loss after loud music	0.49	0.31
Tinnitus after loud music	0.09	0.56

*dB* decibel

The resulting *p* values are given. “*” indicates statistical significance (*p* < 0.05)

For the TWA8 = 65 dB cut-off, where the results are the most pronounced, there was no difference in the preferred music genre, usage of headphones/earphones, active noise canceling technology, ambient noise level, and loud noise exposure history.

The TE values (mean 422 ± SD 146) were categorized according to different cut-off points starting from 300 and increasing to 700. The right-skewed normal distribution pattern and the limited number of participants resulted in an uneven distribution between the two sides of some cut-off values. Therefore, limited sub-group sizes reduced the strength of some conclusions (Table 4). TE cut-off values higher than 700 could not be used statistically. The first significant difference occurred in the TE ≥ 600 group, but a more notable distinction was seen in the TE ≥ 700 group (Table 4). Participants with a TE above 700 exhibited significantly worse hearing thresholds at frequencies of 4, 14, and 16 kHz, as well as in the 125–8 kHz and 500–4 kHz averages compared to those with a TE below 700. Even though threshold differences were only apparent in higher TE values, subjective findings such as speech discrimination difficulty in noisy environments, tinnitus, and hearing loss after exposure to loud music could be observed as low as TE ≥ 400 (Table 5).

**Table 4:** The distribution of mean hearing thresholds and daily music exposures (adjusted to an 8-h continuous exposure-TWA) of participants with different total music exposures is provided

	< 600 *n* = 159	≥ 600 *n* = 23	< 700 *n* = 170	≥ 700 *n* = 12
TWA8 score	60.8	67.7	61.1	69.4
125 Hz	7.3	9.1	7.4*	10*
250 Hz	4.6	6.2	4.6	7.1
500 Hz	6.5	8.26	6.5	10.2
1 kHz	5.5*	8.2*	5.6	10
2 kHz	5.1	7.8	5.3	8.3
4 kHz	3.8	6.6	3.7*	11.3*
8 kHz	5	6.2	4.8	10.4
12.5 kHz	3	3.3	2.6	9
14 kHz	5	6.5	4.4*	16.3*
16 kHz	9.3	12.6	8.8*	21.9*
125-8 kHz	5.4	7.5	5.4*	9.6*
500-4 kHz	5.3	7.7	5.3*	9.9*

Notably, participants below each cut-off value exhibit significantly lower mean TWA8 scores compared to those above it. “*n*” represents the number of participants in each group

*Hz* hertz, *dB* decibels

The symbol “*” indicates statistical significance *(*p < 0.05)

**Table 5:** *p* values of participants are given

	TE ≥ 400	TE ≥ 500	TE ≥ 600
Being warned for speaking loudly	0.41	0.99	0.74
Speech discrimination difficulty in silent environments	0.98	0.21	0.22
Speech discrimination difficulty in noisy environments	0.04*	0.08	0.01*
Difficulty in communicating on the phone	0.09	0.31	0.55
Sound sensitivity after loud music	0.33	0.35	1
Temporary hearing loss after loud music	0.01*	0.04*	0.26
Tinnitus after loud music	0.04*	0.02*	0.38

Participants were categorized based on their total music exposure (TE) levels. The subjective complaints of individuals below the cut-off values were compared to those with higher exposure levels. TE ≥ 700 was appropriate for statistical analysis due to low number of participants

“*” indicates statistical significance (*p* < 0.05)

When TWA8 and TE values were compared, it was seen that for those with TWA8 below 65 dB, the mean TE was 394.8 while it was 477.4 for those with TWA8 > 65 dB (*p* < 0.000). Once the TE values were considered, it was seen that participants with low exposure had significantly lower TWA8 values across all groups than those above the cut-offs (Table 4). Therefore, TWA8 and TE values shared a similar trend.

## Discussion

As music listening habits have become more widespread, particularly at higher volumes and starting from younger ages, recreational music has been recognized as a significant contributing factor to noise-induced hearing loss since the 1950s [5, 7].

Occupational health guidelines recommend keeping noise exposure below TWA8 = 85 dB [4]. However, we propose considering TWA24 instead of TWA8, to account for the ever-increasing noise pollution and recreational music exposure as well as any occupational exposure during the 24 h.

Existing studies examining the effects of recreational music on hearing vary in methodology, making comparisons challenging. Some relied on self-reported questionnaires leading to very rough categorizations of exposure. Sulaiman et al. used a 40-s-long music sample played through insert earphones in a quiet room and asked participants to indicate their preferred music listening volumes [8]. Lee et al. offered participants a choice of nine sample songs from different genres and played them through insert earphones in a testing room with 35 dB ambient noise [3]. In our study, we used the preferred music genres during the “preferred music level tests” but, did not add any ambient noise. The difference in average preferred volumes between electro and classical music reached almost 12 dB, equivalent to nearly a fourfold difference in sound pressure level, confirming that using different genres was the correct choice.

Although methodologies varied to assess the preferred music levels, most studies chose to normalize participants’ daily music exposure to TWA8, allowing for comparisons [2–5, 8]. However, the variables in the cumulative music exposure and the detection of noise exposure primarily relied on retrospective and highly subjective questionnaires, causing high margins of error [1, 2, 7, 8]. Unfortunately, this subjectiveness and whether there is an actual causality between the effect and the outcome of a cross-sectional study could only be overcome with a prospective study design.

The definition of “high-exposure cut-off value” is another subject of debate. For example, Sulaiman et al. used 75 dB, and Lee et al., in which 35 dB ambient noise was added, used 85 dB. In our study, a 65 dB cut-off value revealed the most prominent difference. It’s noteworthy that in our study only one person exceeded TWA8 > 85 dB. Our detection of preferred music levels in a quiet cabin without adding any ambient noise may have led to lower TWA8 values. Therefore, our TWA8 values should not be directly compared to the values of other studies that incorporated ambient noise.

Several studies have employed conventional audiometry to assess hearing across frequencies ranging from 125 to 8000 Hz [2, 9, 10]. Kim et al. observed elevated thresholds in males and participants with higher TEs [10]. On the other hand, Dehnert et al. found no difference between low and high-exposure groups [2]. Sulaiman et al. took a step further by utilizing extended-spectrum audiometry and examined frequencies up to 16 kHz to detect potential early effects of music exposure [8]. Indeed, all the differences they detected were at 8 kHz and above. Interestingly in our study, we detected elevated thresholds in individuals with high daily or total exposure both in low frequencies (< 500 Hz), which is more relevant to everyday hearing, and in very high frequencies (16 kHz).

Our findings support the findings of many other studies, indicating that recreational music exposure may harm hearing in young individuals, depending on its intensity and daily duration [2, 3, 5, 7–9]. More worryingly, detectable hearing losses and subjective complaints started as low as TWA8 = 65 instead of the generally accepted TWA = 85 [3, 4]. Also, participants with TE ≥ 400 reported increased difficulty in communication and demonstrated elevated thresholds at specific frequencies once TE ≥ 700 was reached. This finding is concordant with Sulaiman et al.’s findings, who found elevated high-frequency thresholds (≥ 8 kHz) in participants with TE ≥ 300 [8]**.**

According to our findings, even if TWA8 is kept below 65 dB, it would take approximately 6 years before long-term results appear. We recommend minimizing daily music exposure and adopting any protective measures, such as avoiding listening to music in noisy environments, preferring isolating earbuds or active noise cancelation, and limiting volume to 80% for no more than 90 min [7].

A smart device application monitoring ambient sounds and earphone sounds can precisely detect daily sound exposure, promoting safer listening practices. Future studies using similar technology in prospective cohort designs may elucidate possible the association between music exposure and hearing loss and establish safer limits by tracking real-world music listening habits and sound exposures.

## Conclusion

Recreational music with much lower exposure levels than the universally accepted TWA8 of 85 dB could negatively affect hearing in healthy young adults. Also, long-term negative outcomes of music exposure could appear in 6 years. Therefore, maintaining a maximum TWA8 of 65 dB is recommended.

## Data Availability

Data of this study can be shared upon request.
